# Temporal trend of comorbidity and increasing impacts on mortality, length of stay, and hospital costs of first stroke in Tianjin, North of China

**DOI:** 10.1186/s12962-021-00316-1

**Published:** 2021-09-28

**Authors:** Ruixiao Hao, Xuemei Qi, Xiaoshuang Xia, Lin Wang, Xin Li

**Affiliations:** 1 Department of Neurology, The Second Hospital of Tianjin Medical University, 23 Pingjiang Road, Tianjin, 300211 China; 2 Department of Geriatrics, The Second Hospital of Tianjin Medical University, Tianjin, China

**Keywords:** Stroke, Comorbidity, Mortality, Length of stay, Cost

## Abstract

**Background:**

Stroke patients have a high incidence of comorbidity. Previous studies have shown that comorbidity can impact on the short-term and long-term mortality after stroke.

**Methods:**

Our study aimed to explore the trend of comorbidity among patients with first stroke from 2010 to 2020, and the influence of comorbidity on admission mortality, length of stay and hospitalization costs. 5988 eligible patients were enrolled in our study, and divided into 4 comorbidity burden groups according to Charlson comorbidity index (CCI): none, moderate, severe, very severe. Survival analysis was expressed by Kaplan–Meier curve. Cox regression model was used to analyze the effect of comorbidity on 7-day and in-hospital mortality. Generalized linear model (GLM) was used to analyze the association between comorbidity and hospitalization days and cost.

**Results:**

Compared to patients without comorbidity, those with very severe comorbidity were more likely to be male (342, 57.7%), suffer from ischemic stroke (565, 95.3%), afford higher expense (Midian, 19339.3RMB, IQR13020.7–27485.9RMB), and have a higher in-hospital mortality (60, 10.1%). From 2010 to 2020, proportion of patients with severe and very severe comorbidity increased 12.9%. The heaviest comorbidity burden increased the risk of 7-day mortality (adjusted hazard ratio, 3.51, 95% CI 2.22–5.53) and in-hospital mortality (adjusted hazard ratio, 3.83, 95% CI 2.70–5.45). Patients with very severe comorbidity had a 12% longer LOS and extra 27% expense than those without comorbidity.

**Conclusions:**

Comorbidity burden showed an increasing trend year in past eleven years. The heavy comorbidity burden increased in-hospital mortality, LOS, and hospitalization cost, especially in patients aged 55 years or more. The findings also provide some reference on improvement of health care reform policies and allocation of resources.

**Supplementary Information:**

The online version contains supplementary material available at 10.1186/s12962-021-00316-1.

## Introduction

Stroke is a leading cause of death and disability worldwide with substantial economic burden including hospital costs and post-stroke care [1]. In general, age-standardized death rates and incidence of stroke have a decreased tendency globally except in eastern Asia and southern Africa [2]. Over the past decades, China has undergone a rapid speed of demographic and epidemiological transitions. Instead of infectious diseases, non-communicable diseases have generally increased, such as stroke, chronic obstructive pulmonary disease, lung cancer and so on [3]. Stroke is still a serious public health problem in China with a high level of incidence, prevalence, disability rate, and mortality [3, 4]. Compared with the developed countries, stroke has its own unique epidemiological features based on China’s demographic characteristics [5]. The crude death rate from stroke in China has been increasing faster than other developed countries (such as the United States, the United Kingdom, and Japan) based on the effect of rapid ageing, which putting huge pressure on the public health system [5].

According to the 2010 census [6], China has become one of the fastest aging countries with a proportion of people aged 65 years and older accounting for 8.9% (approximately 119 million) [7]. By 2050, there will be an aging population in China as large as in the developed countries or more. With a rapid speed of aging, age-specific prevalence rate of stroke in our country increased with age no matter sex or types of stroke, with a remarkable increase in people aged 50 years and older [8]. Ischemic stroke is the most common subtype which accounts for 80% of all strokes [4]. Alongside this, the hazard factors of stroke also affect prevalence of many chronic diseases (e.g., myocardial infarction, diabetes, chronic obstructive pulmonary disease, and cancer). Previous studies conducted in developed countries illustrated comorbidity is a strong factor affecting short- and long-term mortality of post-stroke [9, 10], as well as economic burden in admission [11]. However, few studies are devoted to the relationship between stroke and comorbidity in the condition of an aging population with increasing prevalence of comorbidity in China. It is extremely important to provide evidence not only for stroke prevention strategies but also for policy makers to develop tailored strategies for allocation of resources reasonably.

We therefore conducted a population-based study to explore three interesting events among first stroke patients between 2010 and 2020: the development trends of comorbidity; the effect of comorbidity on stroke in-hospital mortality and social and economic burden causing by comorbidity (including length of hospital stay and inpatient costs).

## Materials and methods

### Data sources

The Second Hospital of Tianjin Medical University, located in the north of China, is a general tertiary hospital with a level of medical care. Each patient admitted to our hospital was given a unique admission number based on identification card number, which is convenient to know about the reasons of every visiting hospital. The diagnostic information of each outpatient visit or hospital discharge is recorded according to the *International Classification of Diseases, Ninth Revision, Clinical Modification* (ICD-9-CM) and ICD-10. The record contained the basic information of patients including name, sex, information on birth date, hospital admission numbers, medical insurance methods, length of stay (LOS), hospitalization expenses, one primary diagnosis and one or more secondary diagnoses, and vital status. Because ICD codes can’t distinguish the severity of the disease, we obtained the details of admission patients like the severity of liver and renal function from Electronic Medical Records System. In China, social medical and health payment system is mainly divided into medical insurance and non-medical insurance (i.e., self-payment). The main insurance schemes are the Urban Employee Basic Medical Insurance (UEBMI), the Urban Residence Basic Medical Insurance, and the New Rural Cooperative Medical Scheme, which covered over 92 percentage of the total population by 2011 [12]. The first two programs cover urban working and retired employees and urban residents without formal employment, respectively. The last covers rural residents. The insurance programs in Tianjin are the UEBMI and the Urban Residence Basic Medical Insurance.

### Study cohort

We performed a retrospective analysis on the records of all patients, aged ≥ 18 years, admitted to the department of neurology in our hospital for acute stroke between 1 January 2010 and 31 December 2020. A total of 10,494 patients with the primary diagnosis of stroke were screened using the ICD diagnosis codes with stroke. After excluding the population diagnosed with a history of stroke, eligible patients were enrolled in our study cohort, who hospitalized for first-time stroke.

All the diagnoses of stroke were independently made by two experienced neurologists to ensure the accuracy of diagnoses. All patients performed computed tomography (CT) or magnetic resonance imaging (MRI).

### Comorbidity

We acquired the comorbidities in accordance with primary and secondary diagnoses. To avoid the confusion that whether comorbidities were related to stroke, we eliminated patients diagnosed with the history of cerebrovascular disease and hemiplegia. We calculated the Charlson comorbidity index (CCI), a simple, wildly, and valid method to evaluate the risk of death from comorbid disease [13, 14]. The total CCI scores were the weighted sum of each comorbidity, which was assigned between 1 and 6 points respectively. We divided patients into four groups based on the total scores: none (CCI = 0), moderate (CCI = 1), severe (CCI = 2), and very severe (CCI = 3 or higher) [9].

### Outcome variables

We paid attention to two primer outcomes: the all-cause mortality within 7 days and the all-cause in-hospital mortality. The 7-day mortality rate was equal to the number of deaths over the total number of admitted cases at the 7th day after admission. The in-hospital death rate was calculated as the ratio of death numbers to the total of admitted cases during the period from the admission date to the date of separation (death or discharged alive) [15]. The second outcomes were length of stay expressed in days (in days) and inpatient costs (in RMB). The length of stay (LOS) was defined as the day from the admission to death or discharge.

### Statistical analyses

We performed statistical analyses to summarize basic characteristics of patients. Firstly, the univariate analysis was utilized on baseline characteristics of patients. Categorical variables were presented as count (percentage), and compared using the Pearson χ^2^ test or Fisher’s exact test. Continuous variables with normal distribution were expressed as mean ± standard deviation (SD), and differences in age among different groups were compared with one-way analysis of variance (ANOVA). Continuous variables without normal distribution were expressed as median (interquartile range, IQR), and calculated by Kruskal–Wallis rank test. Kaplan–Meier methods were used for survival curve plotting among different comorbidity burden groups and differences were examined by using the log-rank test. To assess the relationship between comorbidity category and the 7-day as well as inpatient mortality, we performed Cox proportional hazards regression model controlling the confounding factors [16]. Results were presented as hazard ratio (HR) with 95% confidence intervals (CIs) using patients without comorbidity as reference. After performing univariate Cox regression, we further analyzed the covariates with a univariable P value < 0.05 using the multivariable Cox model.

LOS data were over-dispersed distribution and the cost data represented the right-skewed distribution. A generalized linear model (GLM) was conducted to evaluate the LOS and inpatient cost [17]. GLM with a negative binomial distribution was used to analyze LOS, whereas GLM with log link and gamma distribution was performed to assess in-hospital cost differences among groups [17]. All statistical models were conducted by SPSS 25.0 software or GraphPad prism 8.0. Statistical significance was rejection of Null hypothesis with a 2-sided probability value of < 0.05.

## Results

### Patient characteristics

A total of 5988 were included between in 1 January 2010 and 31 December 2020 (Table 1). Of all patients with mean age of 69.3 years, patients aged ≥ 65 years accounted for 63.5%. Most had ischemic stroke (5751, 96%) with mean age 69.5 years, whereas 237 (4%) were hemorrhagic stroke with a younger average age of 66.1 years. The proportion of first stroke was higher in males (58.4%) than in females (41.6%). All patients were divided into four groups in terms of comorbidity categories: none (2878, 48.1%), moderate (1972, 32.9%), severe (545, 9.1%), very severe (593, 9.9%). The patients with heavier comorbidity burden were likely to be elderly male, and with a higher hospitalization cost. It was fortunate that a high proportion of medical insurance system can help reduce the economic burden of illness with basic medical insurance for urban workers as the dominant mode, whereas self-pay accounted for 9.6%. There was no difference in season and week among groups in our study.

**Table 1:** Sociodemographic and clinical characteristics of patients admitted for first stroke

Variables	Total (n = 5988)	Comorbidity category				*P* value
		None (n = 2878)	Moderate (n = 1972)	Severe (n = 545)	Very severe (n = 593)	
Age (mean ± SD)	69.3 ± 12.4	68.3 ± 12.7	69.6 ± 12.1	73.5 ± 11.2	70.2 ± 12.3	< 0.001
Sex, N (%)						
Female	2493 (41.6)	1096 (38.1)	892 (45.2)	254 (46.6)	251 (42.3)	
Age group, N (%)						< 0.001
18–34 years	23 (0.4)	12 (0.4)	5 (0.3)	2 (0.4)	4 (0.7)	
35–44 years	121 (2.0)	74 (2.6)	31 (1.6)	7 (1.3)	9 (1.5)	
45–54 years	627 (10.5)	339 (11.8)	213 (10.8)	23 (4.2)	52 (8.3)	
55–64 years	1412 (23.6)	757 (26.3)	444 (22.5)	83 (15.2)	128 (21.6)	
65–74 years	1431 (23.9)	645 (22.4)	499 (25.3)	142 (26.1)	145 (24.5)	
75–84 years	1757 (29.3)	776 (27.0)	589 (29.9)	202 (37.1)	190 (32.0)	
≥ 85 years	617 (10.3)	275 (9.6)	191 (9.7)	86 (15.8)	65 (11.0)	
Comorbidities, N (%)						
Hypertension	4758 (79.5)	2264 (78.7)	1578 (80.0)	429 (78.7)	487 (82.1)	0.235
Atrial fibrillation or flutter	858 (14.3)	353 (12.3)	285 (14.5)	118 (21.7)	102 (17.2)	< 0.001
Myocardial infarction	365 (6.1)	0 (0)	132 (6.7)	153 (28.1)	80 (13.5)	< 0.001
Congestive heart failure	524 (8.8)	0 (0)	185 (9.4)	157 (28.8)	182 (30.7)	< 0.001
Peripheral vascular disease	256 (4.3)	0 (0)	76 (3.9)	68 (12.5)	112 (18.9)	< 0.001
Dementia	86 (1.4)	0 (0)	52 (2.6)	27 (5.0)	7 (1.2)	< 0.001
Chronic pulmonary disease	48 (0.8)	0 (0)	20 (1.0)	17 (3.1)	11 (1.9)	< 0.001
Connective tissue disease	93 (1.6)	0 (0)	52 (2.6)	23 (4.2)	18 (3.0)	< 0.001
Ulcer disease	106 (1.8)	0 (0)	46 (2.3)	33 (6.1)	27 (4.6)	< 0.001
Mild liver disease	59 (1.0)	0 (0)	22 (1.1)	20 (3.7)	17 (2.9)	< 0.001
Diabetes without end-organ damage	2084 (34.8)	0 (0)	1387 (70.3)	268 (49.2)	429 (72.3)	< 0.001
Diabetes with end-organ damage	223 (3.7)	0 (0)	0 (0)	0 (0)	223 (37.6)	< 0.001
Moderate to severe renal disease	228 (3.8)	0 (0)	0 (0)	71 (13.0)	157 (26.5)	< 0.001
Nonmetastatic solid tumor	201 (3.4)	0 (0)	0 (0)	88 (16.1)	113 (19.1)	< 0.001
Leukemia	7 (0.1)	0 (0)	0 (0)	2 (0.4)	5 (0.8)	< 0.001
Lymphoma	3 (0.1)	0 (0)	0 (0)	1 (0.2)	2 (0.3)	0.003
Moderate to severe liver disease	74 (1.2)	0 (0)	0 (0)	0 (0)	74 (12.5)	< 0.001
Metastatic cancer	16 (0.3)	0 (0)	0 (0)	0 (0)	16 (2.7)	< 0.001
Stroke type, N (%)						< 0.001
Ischemic stroke	5751 (96.0)	2749 (95.5)	1924 (97.6)	513 (94.1)	565 (95.3)	
Hemorrhagic stroke	237 (4.0)	129 (4.5)	48 (2.4)	32 (5.9)	28 (4.7)	
Season, N (%)						0.263
Spring	1481 (24.7)	707 (24.6)	518 (26.3)	133 (24.4)	123 (20.7)	
Summer	1519 (25.4)	756 (26.3)	475 (24.1)	136 (25.0)	152 (25.6)	
Fall	1484 (24.8)	716 (24.9)	480 (24.3)	136 (25.0)	152 (25.6)	
Winter	1504 (25.1)	699 (24.3)	499 (25.3)	140 (25.7)	166 (28.0)	
Week, N (%)						0.464
Weekend	1625 (27.1)	796 (27.7)	510 (25.9)	155 (28.4)	164 (27.1)	
Weekday	4363 (72.9)	2082 (72.3)	1462 (74.1)	390 (71.6)	429 (72.3)	
Payer, N (%)						< 0.001
Urban employee basic medical insurance	4540 (75.8)	2139 (74.3)	1538 (78.0)	403 (73.9)	460 (77.6)	
Urban residence basic medical insurance	874 (14.6)	422 (14.7)	256 (13.0)	100 (18.3)	96 (16.2)	
Self-pay	571 (9.6)	317 (11.0)	178 (9.0)	42 (7.7)	37 (6.2)	
COST median (IQR)	15,151.7 (10,649.7–22,584.9)	14,160.4 (10,020.2–21,148.3)	15,243.5 (11,074.0–22,610.8)	17,936.7 (11,980.8–25,980.4)	19,339.3 (13,020.7–27,485.9)	< 0.001
In-hospital mortality, N (%)	249 (4.2)	65 (2.3)	73 (3.7)	51 (9.4)	60 (10.1)	< 0.001
Admission year, N (%)						< 0.001
2010	397 (6.6)	217 (7.5)	144 (7.3)	23 (4.2)	13 (2.2)	
2011	517 (8.6)	261 (9.1)	206 (10.4)	34 (6.2)	16 (2.7)	
2012	676 (11.3)	356 (12.4)	238 (12.1)	47 (8.6)	35 (5.9)	
2013	635 (10.6)	336 (11.7)	211 (10.7)	53 (9.7)	37 (6.2)	
2014	470 (7.8)	217 (7.5)	170 (8.6)	48 (8.8)	35 (5.9)	
2015	512 (8.6)	241 (8.4)	156 (7.9)	45 (8.3)	70 (11.8)	
2016	557 (9.3)	257 (8.9)	157 (8.0)	50 (9.2)	93 (15.7)	
2017	522 (8.7)	244 (8.5)	152 (7.7)	46 (8.4)	80 (13.5)	
2018	584 (9.8)	266 (9.2)	189 (9.6)	68 (12.5)	61 (10.3)	
2019	617 (10.3)	283 (9.8)	192 (9.7)	70 (12.8)	72 (12.1)	
2020	499 (8.3)	200 (6.9)	157 (8.0)	61 (11.2)	81 (13.7)	

The tendency of comorbidity state and different age groups by calendar year was visually displayed based on the proportion of first stroke (Additional file 1: Figure S1). From 2010 to 2020, the proportion of patients without comorbidity decreased 14.6 percentage points (from 54.7% to 40.1%), while there was a noticeable 12.9 percent increase in patients with very severe comorbidity (from 3.3 to 16.2%) (Additional file 1: Table S1). Proportions of patients aged 55–84 years were higher far more than any other group. Among those, the elderly in the 65–84 years age group almost account for approximately 50%, and the proportion of the oldest age group (≥ 85 years) in 2020 increased 8.3% compared to 2010 (Additional file 1: Table S1). Although male was predominated, there was no sex difference among different year groups (Additional file 1: Table S1).

### Mortality

There was a total of 249 (4.2%) death in the hospital, with 50.2% (n = 125) of women. For in-hospital mortality, the age of the patients was 78.4 ± 9.6 years with 3.9% (223/5528) in ischemic stroke and 69.7 ± 14.9 years with 11.0% (26/211) in hemorrhage stroke. In-hospital mortality in patients with very severe comorbidity was the most (n = 60; 10.1%), those without comorbidity the least (n = 65; 2.3%). The Kaplan–Meier survival curves showed that the in-hospital mortality was higher among patients with severe and very severe comorbidity than patients without comorbidity (Additional file 1: Figure S2a). And the same tendency was observed in the 7-day mortality (Additional file 1: Figure S2b).

Cox regression analysis identified several risk factors of in-hospital mortality (Additional file 1: Table S2). Model 1 was adjusted for age and sex. Model 2 was adjusted for sex, age, season, stroke types. The two models illustrated the consistent result that patients with any comorbidity category had a higher risk of in-hospital mortality compared to those without comorbidity, with the highest risk among those with very severe comorbidity (adjusted hazard ratio in model 2, HR = 3.83, 95% CI, 2.70–5.45) (Table 2). Similar methods to the in-hospital mortality rate (Additional file 1: Table S3), the tendency of increased risk appeared only in patients with severe and very severe comorbidity in 7-day mortality (Table 2).

**Table 2:** Comparison of mortality risks between patients admitted for first stroke with different comorbidity categories

	Univariable analysis		Multivariable analysis: Model 1		Multivariable analysis: Model 2	
	HR (95% CI)	*P* value	aHR^a^ (95% CI)	*P* value	aHR^a^ (95% CI)	*P* value
In-hospital mortality						
None	1.00		1.00		1.00	
Moderate	1.53 (1.10–2.14)	0.013	1.48 (1.06–2.07)	0.022	1.51 (1.08–2.11)	0.016
Severe	3.50 (2.42–5.07)	< 0.001	2.88 (1.98–4.17)	< 0.001	2.88 (1.98–4.17)	< 0.001
Very severe	3.95 (2.78–5.62)	< 0.001	3.84 (2.70–5.46)	< 0.001	3.83 (2.70–5.45)	< 0.001
7-day mortality						
None	1.00		1.00		1.00	
Moderate	1.24 (0.80–1.93)	0.330	1.48 (1.06–2.07)	0.448	1.22 (0.79–1.90)	0.369
Severe	3.61 (2.52–5.78)	< 0.001	2.88 (1.98–4.17)	< 0.001	2.96 (1.84–4.76)	< 0.001
Very severe	3.77 (2.40–5.94)	< 0.001	3.84 (2.70–5.46)	< 0.001	3.51 (2.22–5.53)	< 0.001

*aHR* adjusted hazard ratio, *HR* hazard ratio

^a^Model 1: aHR was calculated with adjustments for age and sex; Model 2: aHR was calculated with adjustments for age, sex, season, stroke type

For patients with ischemic stroke, the increased in-hospital and 7-day death risk among comorbidity groups was consistent with those with stroke (Table 3). Different from ischemic stroke, the increased risk only occurred to those with severe and very severe comorbidity when compared to those without comorbidity in hemorrhage stroke. When patients without comorbidity were as reference, the hazard ratio is higher in hemorrhage stroke than ischemic stroke (Table 3). We further conducted an age-stratified analysis, severe and very severe comorbidity burden had the strongest effect on in-hospital mortality in the elder groups with age ≥ 55 years (Additional file 1: Table S4). Also, season is a significant risk factor of in-hospital death with the highest inpatient mortality in winter. We further stratified seasons and found that the heaviest comorbidity burden was related to in-hospital death in each stratify (Additional file 1: Table S5).

**Table 3:** Comparison of mortality risks between patients admitted for different stroke type with different comorbidity categories

Comorbidity burden^a^	In-hospital Mortality				7-day Mortality			
	Univariable Analysis		Multivariable Analysis		Univariable Analysis		Multivariable Analysis	
	HR (95% CI)	*P* value	aHR (95% CI)	*P* value	HR (95% CI)	*P* value	aHR (95% CI)	*P* value
Ischemic stroke								
None	1.00				1.00		1.00	
Moderate	1.62 (1.14–2.30)	0.007	1.57 (1.11–2.23)	0.012	1.34 (0.84–2.13)	0.215	1.27 (0.80–2.02)	0.310
Severe	3.38 (2.27–5.04)	< 0.001	2.68 (1.79–4.00)	< 0.001	3.51 (2.10–5.87)	< 0.001	3.32 (2.02–5.45)	< 0.001
Very severe	3.95 (2.71–5.75)	< 0.001	3.86 (2.65–5.61)	< 0.001	3.59 (2.18–5.89)	< 0.001	3.32 (2.02–5.45)	< 0.001
Hemorrhagic stroke								
None	1		1		1.00		1.00	
Moderate	0.98 (0.26–3.70)	0.975	0.92 (0.24–3.49)	0.904	0.88 (0.18–4.38)	0.880	0.80 (0.16–4.00)	0.790
Severe	3.91 (1.45–10.56)	0.007	3.97 (1.47–10.70)	0.006	3.49 (1.07–11.44)	0.039	3.51 (1.07–11.52)	0.038
Very severe	3.88 (1.40–10.76)	0.009	3.95 (1.42–10.99)	0.008	4.78 (1.54–14.84)	0.007	4.88 (1.57–15.14)	0.006

*aHR* adjusted hazard ratio, *HR* hazard ratio

^a^Four categories of comorbidity were defined based on Charlson Comorbidity Index scores of 0 (none), 1 (moderate), 2 (severe), and 3 or more (very severe)

**Fig. 1:**
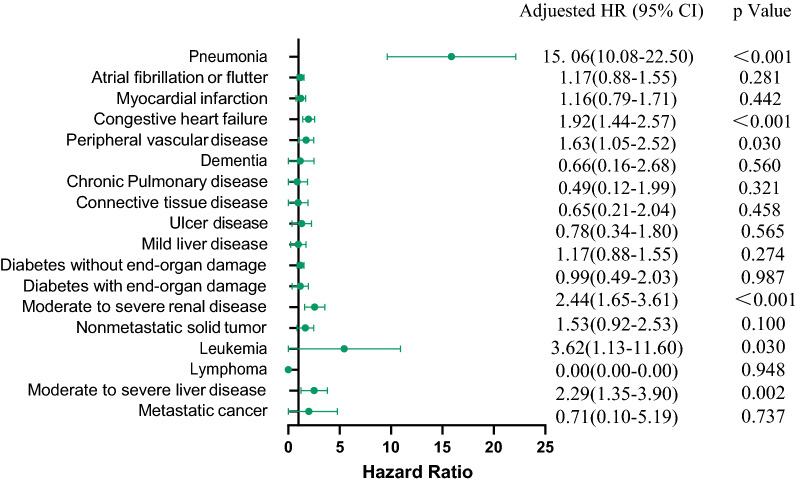
In-hospital mortality associated with individual comorbidities and complications after first-time hospitalization for stroke

The risk of in-hospital death influenced by individual disease in detail was presented in Fig. 1. It was pneumonia, congestive heart failure, peripheral vascular disease, moderate to severe renal and liver disease that were strongly associated with in-hospital mortality. The in-hospital mortality with pneumonia was 15 times higher than without pneumonia. The inpatient mortality was increased 2.3-fold for moderate to severe renal and liver disease. However, there were no connection between the inpatient death risk and the other comorbidity factors such as myocardial infarction, atrial fibrillation or flutter, chronic pulmonary disease, and diabetes with or without end-organ damage. The fatality rate during the first one-week was 2.4% (142/5846). Patients with higher death risk tended to hemorrhage stroke and were more likely to suffer from pneumonia, moderate and severe renal and liver disease, or congestive heart failure (Additional file 1: Table S6).

### Length of stay

The median duration of LOS was 14 (IQR: 11–15) days (Table 1). While the unadjusted median LOS of hemorrhage stroke was 15 (IQR: 12–21) days, longer than that of ischemic patients, which is same as the stroke. Table 4 showed the effect of sociodemographic, clinical characteristics on LOS. There was no difference in LOS between male and female. Those with more comorbidities and older had a tendency of longer LOS. The length of the hospital stay was not affected by admission time whether on the week or in any season. And there was no difference in terms of payment on LOS.

**Table 4:** Estimated length of stay with 95% confidence intervals by patient characteristics

	Model 1		Model 2	
	β (95% CI)	P value	β (95% CI)	P value
Sex				
Male	1.00		–	–
Female	1.02 (0.97–1.07)	0.515	–	–
Age group				
18–34 years	0.79 (0.51–1.22)	0.284	0.77 (0.51–1.19)	0.243
35–44 years	0.80 (0.66–0.99)	0.036	0.80 (0.65–0.98)	0.034
45–54 years	0.84 (0.75–0.95)	0.004	0.83 (0.74–0.93)	0.001
55–64 years	0.85 (0.78–0.94)	0.002	0.85 (0.77–0.93)	0.001
65–74 years	0.90 (0.82–1.00)	0.039	0.89 (0.81–0.98)	0.020
75–84 years	0.92 (0.83–1.01)	0.070	0.90 (0.82–0.99)	0.025
≥ 85 years	1.00		1.00	
Stroke type				
Ischemic stroke	1.00		1.00	
Hemorrhagic stroke	1.19 (1.04–1.36)	0.012	1.18 (1.03–1.35)	0.019
Comorbidity category				
None	1.00		1.00	
Moderate	1.06 (1.00–1.12)	0.055	1.06 (1.00–1.12)	0.070
Severe	1.12 (1.02–1.24)	0.018	1.14 (1.01–1.23)	0.027
Very severe	1.09 (0.99–1.20)	0.058	1.13 (1.03–1.23)	0.013
Admission year				
2010	1.20 (1.04–1.37)	0.011	1.20 (1.04–1.38)	0.010
2011	1.18 (1.04–1.34)	0.012	1.18 (1.04–1.34)	0.013
2012	1.16 (1.03–1.31)	0.016	1.15 (1.02–1.30)	0.026
2013	1.14 (1.01–1.29)	0.038	1.13 (1.00–1.27)	0.055
2014	1.07 (0.94–1.22)	0.307	1.07 (0.94–1.22)	0.315
2015	1.02 (0.95–1.23)	0.258	1.06 (0.93–1.21)	0.347
2016	1.01 (0.90–1.15)	0.793	1.02 (0.90–1.15)	0.816
2017	1.00 (0.87–1.13)	0.916	1.00 (0.88–1.14)	0.968
2018	1.03 (0.91–1.16)	0.667	1.02 (0.90–1.16)	0.745
2019	0.97 (0.86–1.10)	0.658	0.97 (0.86–1.10)	0.634
2020	1.00		1.00	
Payer, N (%)				
Urban employee basic medical	1.01 (0.92–1.10)	0.890	–	–
Urban residence basic medical insurance	0.94 (0.84–1.06)	0.372	–	–
Self-pay	1.00		–	–
Season, N (%)				
Spring	1.05 (0.97–1.13)	0.235	–	–
Summer	1.04 (0.97–1.12)	0.303	–	–
Fall	1.05 (0.97–1.13)	0.232	–	–
Winter	1.00		–	–
Week, N (%)				
Weekend	0.97 (0.91–1.03)	0.272	–	–
Weekday	1.00		–	–

Model 1: unadjusted length of stay; Model 2: length of stay after adjusting age, sex, stroke types, calendar year, comorbidity categories

To further control confounding factors, we conducted the multivariate analysis, and adjusted the age, stroke types, comorbidity categories, and admission year. There was also a positive correlation LOS and comorbidity burden. Compared to the elder aged 85 years and more, the younger had less LOS with a statistical significance except for the 18–34 years age group. And the mean LOS in the younger groups was lower by 10%, 11%, 10.2%, 15%, 17%, and 20% for those between 75 and 84 years, 65 and 74 years, 55 and 64 years, 45 and 54 years, and 35 and 44 years, respectively. The LOS of hemorrhagic stroke was higher by 19% than that of ischemic stroke. There is significant 20 percent points decrease of the LOS in calendar year 2020 compared with ten years ago.

### Inpatient costs

The overall unadjusted median hospitalization expense was 15151.7RMB (IQR: 10649.7–22584.9RMB), which was equivalent to $2307.6 (IQR: $1621.9-$3439.7) based on an exchange rate of 6.57 RMB for per United States dollar. No inpatient cost difference was found between ischemic stroke and hemorrhagic stroke (15190.8RMB, IQR: 10696.1–22,563.8RMB vs. 14694.9 RMB, IQR: 10023.1–22997.7RMB). The median of cost in the four comorbidity groups was 14160.4RMB ($2156.6), 15243.5RMB ($2321.5), 17936.7RMB ($2731.8), and 19339.3RMB ($2945.4), respectively (Table 1). Patients with very severe comorbidity spend the most money. The elder aged ≥ 85 years expensed more than the rest any age groups except for the youngest group. Compared to those without comorbidity, it was obvious that patients carried very severe comorbidity needed to spend the extra 27% expense. Admission on weekday had greater hospital expenses than those with weekend admission. And compared to 2020, the mean inpatient cost was higher by 36% for the cost in 2010. Season and medical payment methods had no effect on cost (Table 5).

**Table 5:** Estimated hospital cost with 95% confidence intervals by characteristics

	Model 1		Model 2	
	B (95%CI)	P value	B (95%CI)	P value
Sex				
Male	1.00		1.00	
Female	1.07 (1.04–1.10)	<0.001	1.00 (0.97–1.03)	0.914
Age group				
18–34 years	0.79 (0.62–1.03)	0.077	0.80 (0.63–1.01)	0.061
35–44 years	0.71 (0.63–0.80)	<0.001	0.76 (0.68–0.85)	<0.001
45–54 years	0.67 (0.62–0.71)	<0.001	0.74 (0.69–0.79)	<0.001
55–64 years	0.70 (0.66–0.75)	<0.001	0.75 (0.71–0.79)	<0.001
65–74 years	0.77 (0.73–0.82)	<0.001	0.82 (0.77–0.86)	<0.001
75–84 years	0.82 (0.78–0.87)	<0.001	0.88 (0.83–0.93)	<0.001
≥ 85 years	1.00		1.00	
Comorbidity Category				
None	1.00		1.00	
Moderate	1.12 (1.08–1.16)	<0.001	1.11 (1.07–1.14)	<0.001
Severe	1.36 (1.28–1.43)	<0.001	1.23 (1.16–1.29)	<0.001
Very severe	1.40 (1.33–1.48)	<0.001	1.27 (1.21–1.39)	<0.001
Week				
Weekend	0.96 (0.93–1.00)	0.029	0.96 (0.93–0.99)	0.006
Weekday	1.00		1.00	
Admission year				
2010	0.61 (0.56–0.66)	<0.001	0.64 (0.59–0.69)	<0.001
2011	0.63 (0.58–0.67)	<0.001	0.65 (0.61–0.70)	<0.001
2012	0.53 (0.50–0.57)	<0.001	0.55 (0.52–0.59)	<0.001
2013	0.62 (0.58–0.66)	<0.001	0.65 (0.60–0.69)	<0.001
2014	0.72 (0.67–0.77)	<0.001	0.73 (0.68–0.79)	<0.001
2015	0.76 (0.71–0.82)	<0.001	0.78 (0.73–0.83)	<0.001
2016	0.77 (0.72–0.82)	<0.001	0.78 (0.73–0.83)	<0.001
2017	0.83 (0.78–0.90)	<0.001	0.84 (0.79–0.90)	<0.001
2018	0.98 (0.91–1.05)	0.537	0.99 (0.93–1.06)	0.866
2019	1.00 (0.93–1.07)	0.974	1.01 (0.94–1.07)	0.878
2020	1.00	1.00	1.00	1.00
Stroke type				
Ischemic stroke	1.01 (0.93–1.09)	0.863	–	–
Hemorrhagic stroke	1.00			
Payer				
Urban employee basic medical	1.00 (0.94–1.05)	0.855	–	–
Urban residence basic medical insurance	0.94 (0.88–1.01)	0.073	–	–
Self-pay	1.00			
Season				
Spring	1.02 (0.97–1.06)	0.456	–	–
Summer	1.02 (0.98–1.07)	0.351	–	–
Fall	1.05 (1.01–1.10)	0.028	–	–
Winter	1.00			

Model 1: unadjusted in-hospital cost; Model 2: in-hospital cost after adjusting age, sex, week, calendar year, comorbidity category

## Discussion

This study first evaluated 11-year trends in first-time stroke with comorbidity, and the effect of comorbidity on mortality, length of stay, and hospital cost from 2010 to 2020 in Tianjin, north of China. We have three main foundings: the age group of first stroke patients was concentrated from 55 to 84 years old during the eleven years, and the population of the old elderly (≥ 85 years old) was increasing with the deepening of aging society; there was a decreased trend of those with no and moderate comorbidity, and an increased tendency of patients with severe and very severe comorbidity; those with severe and very severe comorbidity had higher in-hospital and 7-day mortality, longer LOS and more heavy economic burden, especially in the patients aged 55 to 65 years.

We observed that tendency of comorbidity increasing and aging populations was in line with the study in a developed country of Denmark [9, 10]. While a more serious aging trend in Denmark was observed with the predominance of first-stroke people aged ≥ 70 years (almost 63%), and patients aged 65 years old was the dominant for those with ischemic stroke. Therefore, it was important to pay attention to impact of comorbidity on stroke in an aging society. Although less attention was paid to comorbidity, there were several previous studies in other countries devoted to comorbidity. Higher CCI scores were generally associated with worse function outcome at hospital discharge and greater 1-year mortality of stroke [14, 18]. Some national studies with a large sample size concluded that comorbidity was a strong prognosis predicted factor for not only short-term prognosis, but also 5-year mortality regardless of stroke subtype [9, 19].

A cohort study in Australia divided 776 stroke patients into high and low CCI scores group and found that a higher CCI score as a risk factor increased in-hospital mortality, LOS, and inpatient cost [11], which was consistent with our study. Different from those, the mean age in our study was younger (69.3 years vs 80.1 years), LOS were longer (14 days vs 5.44 days), and mortality in heavy comorbidity burden was lower (19.5% vs 22.1%). The reason for differences may be from the different regions, degree of social aging, national medical development levels, medical insurance policies, and sample size. Compared to the developing countries, Australia had the deeper degree of social aging and better health-care systems. Furthermore, patients aged 80 years old or older had more comorbidities and higher mortality than in those younger than 80 years [20], which further supported that our mortality rate is slightly lower than Australia’s. However, different sample size may be contributed to the results differences, with 5988 patients in our patients larger than 776 population in theirs.

First stroke inpatient mortality in our study was lower than the national study in our country based on community and a sample size of 0.5 million adults (4.2% vs 11%) [4]. For one thing, the latter study based on the big data had more regional diversity, younger population (59.3 vs 69.3 years), and higher proportion of hemorrhage stroke than ours (18% vs 4%). For another thing, the national data estimated the 28-day mortality, while in-hospital mortality in ours. Post-hospital death events may result in the increased mortality. Besides, the big data supported that mortality of hemorrhage stroke was higher than that of ischemic stroke, with the ratio of 11% higher than 3% in our study [4]. A nationwide inpatient data from America also reported that more comorbidities and older age were independently associated with in-hospital mortality [21]. Other studies found that women were related to the increased risk of in-hospital death [21, 22]. In our study, there was no significance in the multivariable analysis, the differences of results may attribute to regional, racial differences, and the different sample size.

We further analyzed the relationship between comorbidity and in-hospital mortality by seasonal stratification and found that patients with severe and very severe comorbidity had higher risk than those without comorbidity no matter in which season, which was in line with a published study [23]. Another 5-year hospital-based study on connection between season and stroke reported that stroke case-fatality rate was the highest in the winter especially in aged ≥ 65 years [24]. While the seasonality of 7-day mortality was never seen in our study, we made an assumption that there was a time lag effect of mortality. It is reported that pneumonia had a higher prevalence in winter [25], and recent infection increased the mortality of stroke [26], which may explain the phenomenon of seasonal difference in our study with the older patients with exist of higher proportion of pneumonia.

After exploring in-hospital mortality associated with individual comorbidities in patients with first stroke, we found that patients with pneumonia occupied first place (HR, 15.06, 95% CI 10.08–22.50, *P* < 0.001), followed by moderate to severe renal (HR, 2.47, 95% CI 1.50–4.08, *P* < 0.001) and moderate to severe liver disease (HR, 2.58, 95% CI 1.32–5.08, *P* = 0.006). The pneumonia may result from dysphagia leading to aspiration pneumonia, acroparalysis leading to long time of stay in bed and hypostatic pneumonia, and climate change in different season leading to respiratory infection. An England study showed that the aspiration pneumonia had a higher short-term mortality than those without aspiration pneumonia [27]. It was validated effective and practicable to perform an early dysphagia screening by neurologist, speech–language therapists, or well-trained nurses [28]. The results of several studies were consistent with our findings for association between in-hospital mortality and kidney dysfunction on admission [29, 30] and liver dysfunction [31]. Therefore, these results remind us that patients with moderate to severe renal and liver dysfunction on admission and dysphagia need to be given targeted intervention strategies to improve their prognosis on discharge, especially the reasons resulting in pneumonia.

Different from previous studies [32–34], inpatient cost had no difference between ischemic and hemorrhage stroke in our study. This may be because conservative medical treatment without surgery in our neurology department. The older with heavy comorbidity burden tended to spend more money and experience longer LOS, which may be caused by that the older needed to pay money and time for the treatment of comorbidities and complications, such as pneumonia, abnormal renal and liver function. And our study demonstrated above hypothesis from a different angle that hospital cost become more higher with the increasing comorbidity burden from 2010 to 2020. Considering the clinical and economic impact among patients with first stroke with different comorbidity categories, especially in the elder with heavy comorbidity burden, the clinical physicians should systematically summarize the impact of age, sex, primary stroke disease, and comorbidity burden calculated by Charlson's comorbidity index.

In addition, the implications of this finding for improving public health insurance and medical services are substantial [35]. Chinese government is embarking on the health care system reform, including the expansion of social health insurance, reform of public hospitals, and strengthening of primary care [36]. As a public hospital, the Second Hospital of Tianjin Medical University effectively implements the national medical reform policies. Diagnosis Related Group System (DRGs) was developed by the Yale Center for Health Studies for a classification of inpatient resource use [37], which is encouraged as the mainstream payment method in public hospitals. At the same time, our hospital has cooperated with primary care institutions to screen the population for stroke risk factors including chronic diseases and guiding disease management.

However, due to restraining factors such as different affordability levels and lack in policy coverage, there are still some restrictions in the protection function of national medical insurance system. Commercial health insurance as an important supplementary form could play a part in risk protection. In China, the degree of participation of commercial health insurance is not very optimistic [38]. There are significant differences between the urban and the rural due to the limitations of funding and education and inadequate publicity in the rural regions [38]. Therefore, increasing the degree of participation of commercial especially for the rural residents can facilitate the establishment of a multi-tiered security system and improvement of a unified national social insurance public service platform.

Several limitations of our study should be acknowledged. First, we conducted the analysis on our admission data according to ICD codes. It is possible that the exist of coding errors or omissions of diagnoses and complications resulted inaccurate classification of comorbidity. Secondly, some important covariates was unavailable, such as body mass index (BMI), personal history (smoking and drinking), subtypes and severity of stroke, thrombolytic therapy, drugs for comorbidities and laboratory results. Thirdly, to facilitate the analysis of in-hospital mortality, we assumed that discharged patients were still alive during the study period [39]. Fourth, although the average LOS was 14 days according to the limitation of medical insurance policy, a longer hospital stay was still required among patients with heavy comorbidity burden. Finally, there was a selection bias considering that our study was a retrospective study at a single center, which may limit the generalisability of our findings. Despite these limitations, our study has its own strength and important implications. The results of a large sample from a comprehensive hospital are representative in Tianjin, and in north of China to some extent due to the similar climate, diet and lifestyle.

## Conclusions

Patients with comorbidity were increasing in number, and comorbidity burden was a strong predicted factor for in-hospital mortality, LOS and inpatient cost, especially in patients aged 55 years or more. The findings also provide some reference on improvement of health care reform policies and allocation of resources.

## Supplementary Information

**Additional file 1: Table S1.** Trends among age, sex, comorbidity of first-stroke population and calendar years. **Table S2.** Cox Regression Analysis of In-hospital Death-Related Risk Factors in First-time Stroke Patients. **Table S3.** Cox Regression Analysis of 7-day Death-Related Risk Factors in First-time Stroke Patients. **Table S4.** Comparison of In-hospital Mortality Risks Between Patients Admitted for First Stroke with Different Comorbidity Categories Using Age-stratified Analysis. **Table S5.** Comparison of In-hospital Mortality Risks Between Patients Admitted for First Stroke with Different Comorbidity Categories Using Season-stratified Analysis. **Table S6.** 7-day Mortality Rate Ratio Associated with Individual Comorbidities After First Time Hospitalization for Stroke. **Figure S1.** Figure shows comorbidity trends of first-stroke population and calendar years (a); figure shows age trends of first-stroke population and calendar years (b). **Figure S2.** Kaplan–Meier survival curves for in-hospital mortality (a) and 7-day mortality (b).

## Data Availability

The datasets used or analysed during the current study are available from the corresponding author on reasonable request.
