# Supplementary material for: Temporal trend of comorbidity and increasing impacts on mortality, length of stay, and hospital costs of first stroke in Tianjin, North of China
Source: Cost Eff Resour Alloc. 2021 Sep 28;19:63. doi: 10.1186/s12962-021-00316-1 (PMC8477574; doi:10.1186/s12962-021-00316-1)
Supplement: Supplementary file 1 — Additional file 1: Table S1. Trends among age, sex, comorbidity of first-stroke population and calendar years. Table S2. Cox Regression Analysis of In-hospital Death-Related Risk Factors in First-time Stroke Patients. Table S3. Cox Regression Analysis of 7-day Death-Related Risk Factors in First-time Stroke Patients. Table S4. Comparison of In-hospital Mortality Risks Between Patients Admitted for First Stroke with Different Comorbidity Categories Using Age-stratified Analysis. Table S5. Comparison of In-hospital Mortality Risks Between Patients Admitted for First Stroke with Different Comorbidity Categories Using Season-stratified Analysis. Table S6. 7-day Mortality Rate Ratio Associated with Individual Comorbidities After First Time Hospitalization for Stroke. Figure S1. Figure shows comorbidity trends of first-stroke population and calendar years (a); figure shows age trends of first-stroke population and calendar years (b). Figure S2. Kaplan–Meier survival curves for in-hospital mortality (a) and 7-day mortality (b). [file 12962_2021_316_MOESM1_ESM.docx]

ADDITIONAL MATERIAL

Additional Tables S1–S6

Table S1. Trends among age, sex, comorbidity of first-stroke population and calendar years.

Table S2. Cox Regression Analysis of In-hospital Death-Related Risk Factors in First-time Stroke Patients.

Table S3. Cox Regression Analysis of 7-day Death-Related Risk Factors in First-time Stroke Patients.

Table S4. Comparison of In-hospital Mortality Risks Between Patients Admitted for First Stroke with Different Comorbidity Categories Using Age-stratified Analysis.

Table S5. Comparison of In-hospital Mortality Risks Between Patients Admitted for First Stroke with Different Comorbidity Categories Using Season-stratified Analysis.

Table S6. 7-day Mortality Rate Ratio Associated with Individual Comorbidities After First Time Hospitalization for Stroke.

Online Figures S1–S2

Figure S1. Figure shows comorbidity trends of first-stroke population and calendar years (a); figure shows age trends of first-stroke population and calendar years (b).

Figure S2. Kaplan-Meier survival curves for in-hospital mortality (a) and 7-day mortality (b).

Table S1. Trends among age, sex, comorbidity of first-stroke population and calendar years

|  | 2010 | 2011 | 2012 | 2013 | 2014 | 2015 | 2016 | 2017 | 2018 | 2019 | 2020 | *P* value |
| --- | --- | --- | --- | --- | --- | --- | --- | --- | --- | --- | --- | --- |
| Age groups, N (%) |  |  |  |  |  |  |  |  |  |  |  | ＜0.001 |
| 18-34 years | 1(0.3) | 3(0.6) | 4(0.6) | 2(0.3) | 1(0.2) | 1(0.2) | 2(0.4) | 3(0.6) | 0(0) | 4(0.6) | 2(0.4) |  |
| 35-44 years | 8(2.0) | 9(1.7) | 3(0.4) | 18(2.8) | 11(2.3) | 13(2.5) | 12(2.2) | 10(1.9) | 16(2.7) | 14(2.3) | 7(1.4) |  |
| 45-54 years | 46(11.6) | 56(10.8) | 94(13.9) | 77(12.1) | 53(11.3) | 39(7.6) | 68(12.2) | 64(12.3) | 48(8.2) | 42(6.8) | 40(8.0) |  |
| 55-64 years | 105(26.4) | 120(23.2) | 138(20.4) | 151(23.7) | 114(24.3) | 133(26.0) | 142(25.5) | 121(23.2) | 140(24.0) | 133(21.6) | 115(23.0) |  |
| 65-74 years | 90(22.7) | 107(20.7) | 176(26.0) | 145(22.8) | 112(23.8) | 123(24.0) | 133(23.9) | 106(20.3) | 149(25.5) | 154(25.0) | 136(27.3) |  |
| 75-84 years | 129(32.5) | 182(35.2) | 212(31.4) | 192(30.1) | 124(26.4) | 160(31.3) | 132(23.7) | 146(28.0) | 168(28.8) | 177(28.7) | 135(27.1) |  |
| ≥ 85 years | 18(4.5) | 40(7.7) | 49(7.2) | 52(8.2) | 55(11.7) | 43(8.4) | 68(12.2) | 72(13.8) | 63(10.8) | 93(15.1) | 64(12.8) |  |
| Sex, N (%) |  |  |  |  |  |  |  |  |  |  |  | 0.111 |
| Male | 237(59.7) | 310(60.0) | 413(61.1) | 393(61.7) | 269(57.2) | 274(53.5) | 334(60.0) | 309(59.2) | 327(56.0) | 341(55.3) | 288(57.7) |  |
| Female | 160(40.3) | 207(40.0) | 263(38.9) | 244(38.3) | 201(42.8) | 238(46.5) | 223(40.0) | 213(40.8) | 257(44.0) | 276(44.7) | 211(42.3) |  |
| Comorbidity burden, N (%) |  |  |  |  |  |  |  |  |  |  |  | ＜0.001 |
| None | 217(54.7) | 261(50.5) | 356(52.7) | 336(52.7) | 217(46.2) | 241(47.1) | 257(46.1) | 244(46.7) | 266(45.5) | 283(45.9) | 200(40.1) |  |
| Moderate | 144(36.3) | 206(39.8) | 238(35.2) | 211(33.1) | 170(36.2) | 156(30.5) | 157(28.2) | 152(29.1) | 189(32.4) | 192(31.1) | 157(31.5) |  |
| Severe | 23(5.8) | 34(6.6) | 47(7.0) | 53(8.3) | 48(10.2) | 45(8.8) | 50(9.0) | 46(8.8) | 68(11.6) | 70(11.3) | 61(12.2) |  |
| Very severe | 13(3.3) | 16(3.1) | 35(5.2) | 37(5.8) | 35(7.4) | 70(13.7) | 93(16.7) | 80(15.3) | 61(10.4) | 72(11.7) | 81(16.2) |  |

Table S2. Cox Regression Analysis of In-hospital Death-Related Risk Factors in First-time Stroke Patients

|  | Univariable Analysis |  | Multivariable Analysis 1 |  | Multivariable Analysis 2 |  |
| --- | --- | --- | --- | --- | --- | --- |
|  | HR (95% CI) | *P* value | HR (95% CI) | *P* value | HR (95% CI) | *P* value |
| **Age** | 1.06(1.05-1.07) | ＜0.001 | 1.06(1.04-1.07) | ＜0.001 | 1.06(1.04-1.07) | ＜0.001 |
| **Female** | 1.34(1.05-1.72) | 0.021 | 1.05(0.81-1.34) | 0.730 | 1.02(0.79-1.31) | 0.874 |
| **Comorbidity burden** |  |  |  |  |  |  |
| None | 1.00 |  | 1.00 |  | 1.00 |  |
| Moderate | 1.53(1.10-2.14) | 0.013 | 1.48(1.06-2.07) | 0.022 | 1.51(1.08-2.11) | 0.016 |
| Severe | 3.50(2.42-5.07) | ＜0.001 | 2.88(1.98-4.17) | ＜0.001 | 2.88(1.98-4.17) | ＜0.001 |
| Very severe | 3.95(2.78-5.62) | ＜0.001 | 3.84(2.70-5.46) | ＜0.001 | 3.83(2.70-5.45) | ＜0.001 |
| **Stroke type** |  |  |  |  |  |  |
| Ischemic stroke | 1.00 |  |  |  | 1.00 |  |
| Hemorrhagic stroke | 2.24(1.49-3.38) | ＜0.001 |  |  | 2.56(1.70-3.87) | ＜0.001 |
| **Season** |  |  |  |  |  |  |
| Spring | 0.64(0.46-0.89) | 0.008 |  |  | 0.67(0.48-0.94) | 0.019 |
| Summer | 0.58(0.41-0.82) | 0.002 |  |  | 0.62(0.44-0.87) | 0.006 |
| Fall | 0.54(0.38-0.76) | ＜0.001 |  |  | 0.52(0.37-0.74) | ＜0.001 |
| Winter | 1.00 |  |  |  | 1.00 |  |
| **Week** |  |  |  |  |  |  |
| weekend | 1.00 |  |  |  | - | - |
| weekday | 0.85(0.65-1.12) | 0.251 |  |  | - | - |

Table S3. Cox Regression Analysis of 7-day Death-Related Risk Factors in First-time Stroke Patients

|  | Univariable Analysis |  | Multivariable Analysis 1 |  | Multivariable Analysis 2 |  |
| --- | --- | --- | --- | --- | --- | --- |
|  | HR (95% CI) | *P* value | HR (95% CI) | *P* value | HR (95% CI) | *P* value |
| Age | 1.05(1.04-1.07) | ＜0.001 | 1.05(1.03-1.06) | ＜0.001 | 1.05(1.03-1.07) | ＜0.001 |
| Female | 1.41(1.02-1.97) | 0.037 | 1.12(0.80-1.57) | 0.504 | 1.10(0.79-1.54) | 0.564 |
| Comorbidity burden |  |  |  |  |  |  |
| None | 1.00 |  | 1.00 |  | 1.00 |  |
| Moderate | 1.24(0.80-1.93) | 0.330 | 1.48(1.06-2.07) | 0.448 | 1.22(0.79-1.90) | 0.369 |
| Severe | 3.61(2.52-5.78) | ＜0.001 | 2.88(1.98-4.17) | ＜0.001 | 2.96(1.84-4.76) | ＜0.001 |
| Very severe | 3.77(2.40-5.94) | ＜0.001 | 3.84(2.70-5.46) | ＜0.001 | 3.51(2.22-5.53) | ＜0.001 |
| Stroke type |  |  |  |  |  |  |
| Ischemic stroke | 1.00 |  |  |  | 1.00 |  |
| Hemorrhagic stroke | 3.87(2.39-6.28) | ＜0.001 |  |  | 4.14(2.55-6.73) | ＜0.001 |
| Season |  |  |  |  |  |  |
| Spring | 0.71(0.46-1.10) | 0.127 |  |  | 0.75(0.49-1.17) | 0.206 |
| Summer | 0.47(0.29-0.77) | 0.003 |  |  | 0.50(0.30-0.82) | 0.006 |
| Fall | 0.77(0.50-1.19) | 0.240 |  |  | 0.76(0.50-0.82) | 0.220 |
| Winter | 1.00 |  |  |  | 1.00 |  |
| Week |  |  |  |  |  |  |
| weekend | 1.00 |  |  |  |  |  |
| weekday | 0.82(0.58-1.18) | 0.283 |  |  |  |  |

Table S4. Comparison of In-hospital Mortality Risks Between Patients Admitted for First Stroke with Different Comorbidity Categories Using Age-stratified Analysis

|  | Comorbidity Category | | | | | | | |
| --- | --- | --- | --- | --- | --- | --- | --- | --- |
| Age groups | None | | Moderate | | Severe | | Very Severe | |
|  | HR (95% CI) | *P* value | HR (95% CI) | *P* value | HR (95% CI) | *P* value | HR (95% CI) | *P* value |
| 18-34 years | 1.00 |  | 0.11(-) | 0.738 | 0.11(-) | 0.832 | 0.11(-) | 0.765 |
| 35-44 years | 1.00 |  | - | - | - | - | - | - |
| 45-54 years | 1.00 |  | 0.71(0.06-7.80) | 0.777 | 5.33(0.74-60.17) | 0.176 | 3.25(0.30-35.89) | 0.336 |
| 55-64 years | 1.00 |  | 1.49(0.54-4.13) | 0.439 | 5.08(1.65-15.66) | 0.005 | 6.95(2.74-17.64) | ＜0.001 |
| 65-74 years | 1.00 |  | 1.08(0.44-2.65) | 0.874 | 2.92(1.11-7.70) | 0.030 | 3.71(1.51-9.16) | 0.004 |
| 75-84 years | 1.00 |  | 1.54(0.93-2.54) | 0.092 | 3.23(1.92-5.63) | ＜0.001 | 3.40(1.98-5.84) | ＜0.001 |
| ≥ 85 years | 1.00 |  | 1.87(0.98-3.57) | 0.057 | 2.00(0.93-4.29) | 0.074 | 3.60(1.75-7.41) | 0.001 |

Table S5. Comparison of In-hospital Mortality Risks Between Patients Admitted for First Stroke with Different Comorbidity Categories Using Season-stratified Analysis

|  | In-hospital Mortality | | | |
| --- | --- | --- | --- | --- |
|  | Univariable Analysis |  | Multivariable Analysis |  |
| Spring |  |  |  |  |
| None | 1.00 |  | 1.00 |  |
| Moderate | 1.54(0.76-3.13) | 0.232 | 1.58(0.77-3.21) | 0.211 |
| Severe | 5.22(2.53-10.78) | ＜0.001 | 4.35(2.11-8.98) | ＜0.001 |
| Very severe | 4.09(1.85-9.02) | ＜0.001 | 3.62(1.64-8.02) | 0.002 |
| Summer |  |  |  |  |
| None | 1.00 |  | 1.00 |  |
| Moderate | 1.07(0.53-2.20) | 0.844 | 1.11(0.54-2.28) | 0.768 |
| Severe | 2.43(1.12-5.30) | 0.025 | 1.83(0.84-4.02) | 0.131 |
| Very severe | 2.52(1.20-5.28) | 0.015 | 2.45(1.17-5.12) | 0.017 |
| Fall |  |  |  |  |
| None | 1.00 |  | 1.00 |  |
| Moderate | 1.69(0.78-3.66) | 0.182 | 1.60(0.74-3.47) | 0.231 |
| Severe | 4.29(1.88-9.80) | 0.001 | 3.53(1.54-8.09) | 0.003 |
| Very severe | 4.41(1.98-9.83) | ＜0.001 | 4.20(1.88-9.37) | ＜0.001 |
| Winter |  |  |  |  |
| None | 1.00 |  | 1.00 |  |
| Moderate | 1.79(1.02-3.15) | 0.042 | 1.70(0.97-3.00) | 0.064 |
| Severe | 2.74(1.38-5.43) | 0.004 | 2.43(1.22-4.85) | 0.012 |
| Very severe | 4.51(2.52-8.08) | ＜0.001 | 4.43(2.47-7.95) | ＜0.001 |

Table S6. 7-day Mortality Rate Ratio Associated with Individual Comorbidities After First Time Hospitalization for Stroke

|  | 7-day Mortality | |
| --- | --- | --- |
|  | HR (95% CI) | *P* value |
| Pneumonia | 14.97(9.27-24.18) | ＜0.001 |
| Atrial fibrillation or flutter | 1.17(0.80-1.72) | 0.424 |
| Myocardial infarction | 1.52(0.93-2.48) | 0.095 |
| Congestive heart failure | 1.51(1.01-2.25) | 0.044 |
| Peripheral vascular disease | 1.53(0.80-2.93) | 0.199 |
| Dementia | 1.23(0.30-5.04) | 0.770 |
| Chronic Pulmonary disease | - | 0.971 |
| Connective tissue disease | 1.20(0.38-3.79) | 0.763 |
| Ulcer disease | 1.35(0.54-3.39) | 0.518 |
| Mild liver disease | 1.48(0.59-3.75) | 0.402 |
| Diabetes without end-organ damage | 1.06(0.73-1.54) | 0.753 |
| Diabetes with end-organ damage | 0.83(0.31-2.25) | 0.715 |
| Moderate to severe renal disease | 2.47(1.50-4.08) | ＜0.001 |
| Nonmetastatic solid tumor | 0.59(0.22-1.63) | 0.313 |
| Leukemia | 5.17(1.24-21.58) | 0.024 |
| Lymphoma | 0.00(0.00-0.00) | 0.993 |
| Moderate to severe liver disease | 2.58(1.32-5.08) | 0.006 |
| Metastatic cancer | 0.71(0.10-5.19) | 0.927 |


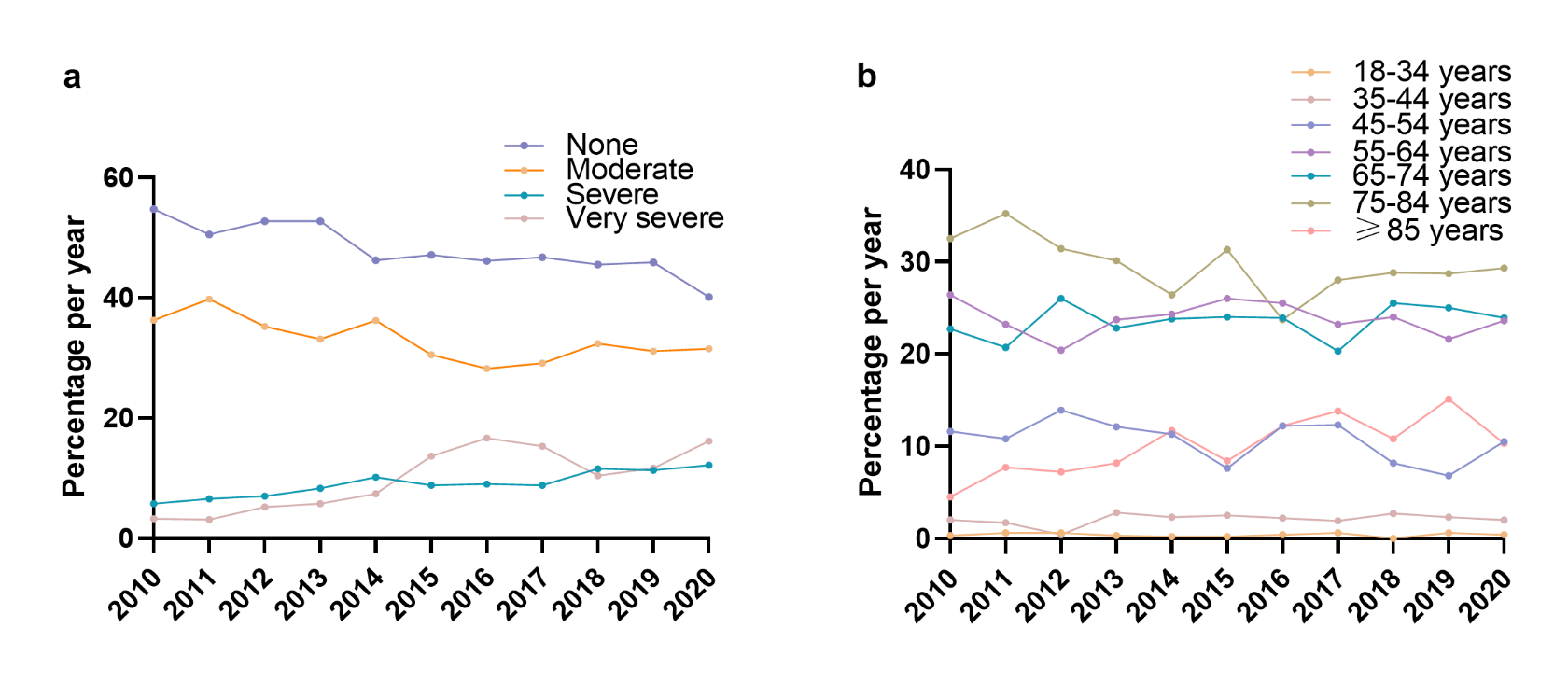


Figure S1. Figure shows comorbidity trends of first-stroke population and calendar years (a); figure shows age trends of first-stroke population and calendar years (b).


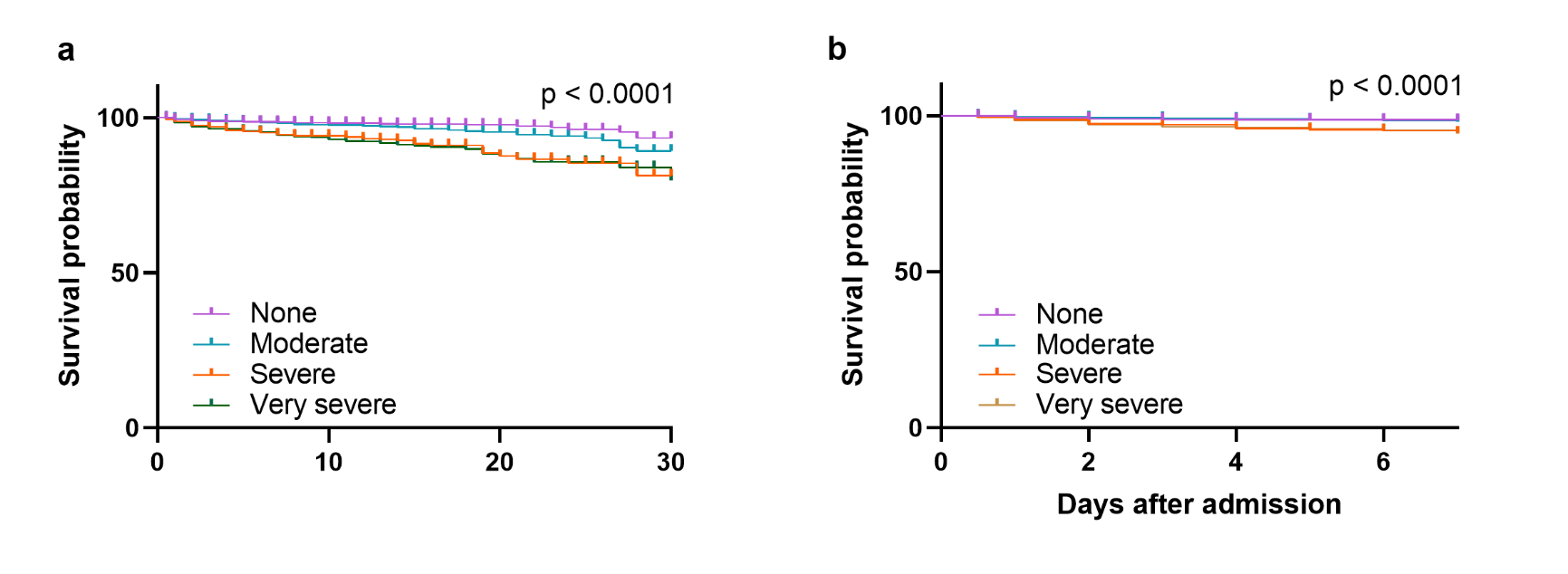


Figure S2. Kaplan-Meier survival curves for in-hospital mortality (a) and 7-day mortality (b).
